# Editorial: Developing research potential in the primary and community-nursing workforce: the impact of a community of practice

**DOI:** 10.1017/S1463423623000543

**Published:** 2023-11-28

**Authors:** Eleanor Hoverd, Joanne Cooper, Sue Shortland, Peter Zeh, Ben Bowers, Lee Tomlinson, Sandra Dyer, Paula Boyer, Jen Charlewood, Andrew Finney

**Affiliations:** 1 Unit of Academic Primary Care, University of Warwick, Warwick Medical School, Coventry, UK; 2 Nottingham University Hospitals NHS Trust, Nottingham, UK; 3 Birmingham City University, Birmingham, UK; 4 Coventry University, Coventry, UK; 5 University of Cambridge, Cambridge, UK; 6 Kent Community Health NHS Foundation Trust, Ashford, UK; 7 Royal Free London NHS Foundation Trust, London, UK; 8 Rotherham NHS Foundation Trust, Rotherham, UK; 9 NHS South of England: NHS England and NHS Improvement South West, Gloucestershire, UK; 10 Keele University School of Nursing and Midwifery, Newcastle-under-Lyme, UK

Nurses are an under-represented discipline in health and care research career pathways, even more so in primary and community settings (Avery *et al.*, 2022). Primary care and community nurses are an extremely diverse workforce, working in distinct specialities supporting patients and their families across the lifespan (NHS, 2023). They have the experiential knowledge required to understand the needs of patients, families and communities, working in partnership with them, to recognise what works and what does not. This unique nature of their roles requires them to build long-term relationships with their patients, liaising across services; they are in an ideal position to develop, support and conduct research, informed by their insights and clinical expertise (DHSC, 2013). However, there are few nurses within these settings who are actively involved in health and care research and even fewer embarking on their own clinical academic careers (Bradbury *et al.*, 2021).

It is over 20 years since Griffiths et al reported that the key barriers to community nurses implementing research into practice have included a lack of time and resources, suggesting that the development of nurses’ critical appraisal skills may support implementation of research, given the time and funding to do so (Griffiths *et al.*, 2001). The UK Clinical Research Collaboration highlighted in their 2007 report, the importance of the development of clinical academic careers for nurses, not only to ensure that people and communities receive the best care and improved outcomes but also to develop the potential of the nursing workforce (UK Clinical Research Collaboration, 2007). The development of a nursing workforce that values research and innovation and has the expertise and the ability to respond to turbulent organisational changes was a key aim (UK Clinical Research Collaboration, 2007). However, barriers to becoming involved in, and developing a clinical academic career in primary care and community settings remain, somewhat due to the historical training and education of nurses not incorporating research as part of the role (Finch, 2009). Despite the numerous barriers that nurses must traverse to achieve a clinical academic career, there are huge benefits to be realised.

Realising nurses’ full potential, from designing to implementing research, is recognised as a vital element in enhancing patient care and maximising the contribution of primary and community nurses to the transformation of services (NHSE, 2021). The significant focus on the development of integrated care systems in the UK, prioritising preventative care for people, carers, communities and families in addition to providing health and social care services, requires an improved evidence base to ensure high-quality care and services are provided (Charles, 2022). Nurses are suitably placed to contribute to this through leading research roles in primary and community settings to improve equity of access for participation in health and care research studies across care pathways and systems. In fact, the value of primary and community care research being led and delivered by nurses must be incorporated as a routine part of advancing practice. This is also vital in developing clinical academic careers and empowering nurses to routinely lead interdisciplinary research. While the development of clinical academic careers is challenging for nurses across settings, this is further compounded for those outside hospital-based care environments due to limited role models, infrastructure, research activity and funding (Bowers, 2018). It is therefore crucial that a focus on developing an organisational culture that values research and innovation, through organisational and strategic leadership, coincides with the development of capability and capacity of the nursing workforce in primary care and community settings (Peckham *et al.*, 2023).

Research-active organisations in the UK have better patient outcomes (Ozdemir *et al.*, 2015). Therefore, research in primary care and the community is likely to be central to improving overall patient care and reducing health inequalities (Ozdemir *et al.*, 2015). Primary care and community nurses understand the significance of a strong evidence base and have been shown to have the knowledge and skills to be able to identify and appraise the evidence, as well as develop their own research (Finney *et al.*, 2020). Yet, challenges to embedding research activity persist. There are perceptions that research is an ‘add on’ role and concerns that a research career lacks clarity or may hinder clinical career progression, or that research can only be led by medical colleagues (Clifford *et al.*, 2021). Such perceptions and the long-term workload pressures in primary and community settings can make it appear almost impossible to participate in health and care research. However, there is a growing interest within the nursing workforce on how to deliver the best health outcomes for patients through research following the publication of the Chief Nursing Officer for England’s strategic plan for research ‘Making research matter’ in 2021, which set out a clear vision for research to be led by nurses (NHSE, 2021).

In response to calls from primary care and community nurses, we established a national community of practice (CoP) *Clinical Academic Careers in Primary Care and Community Nursing* in September 2021. Nurses expressed repeatedly that they need to use the voices and experiences of peers *leading* and *doing* research to inspire others as trailblazers and role models, actively and collectively creating an empowering environment for facilitating the capacity and capability of nurses in research (Avery *et al.*, 2022).

Communities of practice can play a vital role in fostering professional learning (Ikioda & Kendall, 2016, Ikioda *et al.*, 2013, Wenger, 1998). They are an informal way of bringing like-minded peers together who share the same passion or interest and willingness to network and learn how to ‘do it better’ through frequent CoP discussions (Graven & Lerman, 2003). This common interest has shaped the identity of our CoP and has brought with it the desire and motivation for change (Graven & Lerman, 2003). The tacit knowledge within CoPs can be more effective for creating change than any other form of knowledge, as it encompasses the valuable ‘know-how’ that is based on personal and practical experience (Pyrko *et al.*, 2017).

The UK-wide CoP welcomes nurses who are passionate about improving care for patients through conducting their own research and supporting other nurses in primary care and community nursing to become involved in research. The CoP includes a range of diverse roles including general practice nurses, advanced clinical practitioners, community nurses, matrons and consultants, nurse researchers, clinical academics, senior academics, senior managers and national nursing leaders in the NHS. Its aim is to develop and support the research potential of the nursing workforce in primary care and community nursing and utilise the pool of diverse talents to support other nurses interested in becoming nurse researchers. We strive to champion other nurses to become researchers and research leaders.

Since its conception in September 2021, our CoP has connected nurses around the UK in primary care and community nursing with positive benefits. For example, two members have submitted PhD fellowship applications for funding, supported by post-doctoral members of the CoP. We have created a ‘buddying’ approach for those immersed in PhDs within their clinical role and hosted a national virtual conference on 31st March 2022 with over 300 nursing delegates in attendance, to showcase examples of the best nursing research and innovation in primary care and community nursing. Previous evidence demonstrating the success of virtual CoP suggests ensuring adequate support to co-ordinate CoP is in place, as well as providing regular communication to promote engagement (Ikioda & Kendall, 2016). The CoP continues to grow, supporting members at different career stages to release their potential. The influence of the CoP has led to funding from the NHS England General Practice Nurse Programme being allocated by the Southwest Region of England for a General Practice Nursing Research Clinical Fellow to promote nurse-led research and careers.

As a community of influence, we are forging a collaborative approach with other nursing research groups to harness the unique aspects that each group has to offer. The CoP collaborates with other support networks, such as the Queen’s Nursing Institute Community Nursing Research Forum (https://qni.org.uk/nursing-in-the-community/community-nursing-research-forum/) and the National Institute for Health and Care Research School for Primary Care Research (https://www.spcr.nihr.ac.uk/career-development/funding), demonstrating a real need to maximise access for learning and developing research careers and releasing nursing research capacity and potential in these settings (NHSE, 2021, The Queen’s Nursing Institute, 2023, NIHR, 2023).
